# Brown Adipose Tissue in Cetacean Blubber

**DOI:** 10.1371/journal.pone.0116734

**Published:** 2015-02-26

**Authors:** Osamu Hashimoto, Hirofumi Ohtsuki, Takehiko Kakizaki, Kento Amou, Ryo Sato, Satoru Doi, Sara Kobayashi, Ayaka Matsuda, Makoto Sugiyama, Masayuki Funaba, Takashi Matsuishi, Fumio Terasawa, Junji Shindo, Hideki Endo

**Affiliations:** 1 Kitasato University School of Veterinary Medicine, Towada, Aomori 034-8628, Japan; 2 The University Museum, The University of Tokyo, Bunkyo-ku, Tokyo 113-0033, Japan; 3 Faculty of Fisheries Sciences, Graduate School of Fisheries Sciences, Hokkaido University, Hakodate, Hokkaido 041-8611, Japan; 4 Division of Applied Biosciences, Kyoto University Graduate School of Agriculture, Kitashirakawa Oiwakecho, Kyoto 606-8502, Japan; 5 Enoshima Aquarium, Fujisawa, Kanagawa 251-0035, Japan; University of Western Australia, AUSTRALIA

## Abstract

Brown adipose tissue (BAT) plays an important role in thermoregulation in species living in cold environments, given heat can be generated from its chemical energy reserves. Here we investigate the existence of BAT in blubber in four species of delphinoid cetacean, the Pacific white-sided and bottlenose dolphins, *Lagenorhynchus obliquidens* and *Tursiops truncates*, and Dall’s and harbour porpoises, *Phocoenoides dalli* and *Phocoena phocoena*. Histology revealed adipocytes with small unilocular fat droplets and a large eosinophilic cytoplasm intermingled with connective tissue in the innermost layers of blubber. Chemistry revealed a brown adipocyte-specific mitochondrial protein, uncoupling protein 1 (UCP1), within these same adipocytes, but not those distributed elsewhere throughout the blubber. Western blot analysis of extracts from the inner blubber layer confirmed that the immunohistochemical positive reaction was specific to UCP1 and that this adipose tissue was BAT. To better understand the distribution of BAT throughout the entire cetacean body, cadavers were subjected to computed tomography (CT) scanning. Resulting imagery, coupled with histological corroboration of fine tissue structure, revealed adipocytes intermingled with connective tissue in the lowest layer of blubber were distributed within a thin, highly dense layer that extended the length of the body, with the exception of the rostrum, fin and fluke regions. As such, we describe BAT effectively enveloping the cetacean body. Our results suggest that delphinoid blubber could serve a role additional to those frequently attributed to it: simple insulation blanket, energy storage, hydrodynamic streamlining or contributor to positive buoyancy. We believe delphinoid BAT might also function like an electric blanket, enabling animals to frequent waters cooler than blubber as an insulator alone might otherwise allow an animal to withstand, or allow animals to maintain body temperature in cool waters during sustained periods of physical inactivity.

## Introduction

Despite having been first recognized in the mid-16^th^ century [1], only over the past 50 years have the origin and function of brown adipose tissue (BAT) in mammals been subject to intensive investigative research [2]. White adipose tissue (WAT) is mainly involved in the storage and mobilization of energy in the form of triglycerides, whereas BAT specializes in dissipating energy as heat during cold- or diet-induced thermogenesis. BAT is particularly abundant in hibernating animals, such as small rodents, where it is concentrated in small deposits in inter- and subscapular, axillary, perirenal and periaortic regions of the body [2]. It has not been recorded in cetaceans, such as whales, dolphins and porpoises, of which the latter two are hereafter referred to as delphinoids (superfamily Delphinoidea).

The thermogenic function of BAT results from the expression of a series of genes related to a high mitochondrial content, and elevated cellular respiration that is largely uncoupled from ATP synthesis. The uncoupling occurs through expression of uncoupling protein 1 (UCP1), a brown adipocyte-specific mitochondrial protein, that promotes proton leak across the inner mitochondrial membrane in mammals [3].

Activation of the rodent synaptic nervous system by cold exposure or β3 agonist administration can induce UCP1 positive adipocytes within WAT in a process called “browning” [3]. In humans, cold-activated brown adipocytes were observed primarily in the cervical, supraclavicular and paravertebral regions using integrated positron emission tomography-computed tomography (PET-CT) and ^18^F-labelled glucose analogue, fluorodeoxyglucose, as a tracer [4–7]. Although these “brown adipocytes,” referred to as inducible brown fat cells, “brown in white” (brite) adipocytes [8], or beige cells [9], also exhibit UCP1 positive activity, their lineage is distinct from that of classical brown adipocytes (BAT), and their origins are different [10]: brown adipocytes are differentiated from Myf5 positive cells, precursors to skeletal muscle, whereas beige cells are derived from Myf5 negative cells. It is thought that beige cells could be transdifferentiated from white adipocytes [11].

Aquatic mammals such as cetaceans (e.g., whales and dolphins), pinnipeds (e.g., seals) and sirenians (e.g., sea cows) have blubber, a thick subcutaneous fatty deposit that contributes to the storage of metabolic energy [12, 13], hydrodynamics [13–15], positive buoyancy [16–18] and thermal insulation [19–21]. However, despite fat being a poor thermal conductor, blubber alone is unlikely to adequately insulate a cetacean against heat loss in cold conditions [22]. Accordingly, alternate means of thermoregulation might be required or advantageous for aquatic mammals.

We hypothesize the existence of brown or brite/beige adipocytes in cetacean blubber, and propose these cells enable cetaceans to adapt to cold environments. This hypothesis was tested by investigating the expression of adipocytes and UCP1 in blubber samples collected from four delphinoid taxa occurring in the Pacific Ocean: the Pacific white-sided and bottlenose dolphins, *Lagenorhynchus obliquidens* and *Tursiops truncatus*, and Dall’s and harbour porpoises, *Phocoenoides dalli* and *Phocoena phocoena*. Our study reveals that BAT occurs within a layer of blubber and connective tissue that extends almost the entire length of the delphinoid body, possibly enabling these animals to withstand temperatures below those blubber as an insulator alone might otherwise allow, or enabling animals to maintain body temperature in cool waters during periods of physical inactivity.

## Materials and Methods

### Specimens and samples

Samples were obtained from four cetacean species: Pacific white-sided and bottlenose dolphins, and Dall’s and harbour porpoises (Table 1). Animals could have become entangled in the nets up to 48 hours before tissues could be sampled, given nets were typically set for one or two days. Accordingly, the presumed time of death was standardized as two days prior to tissue sampling.

**Table 1:** Biological data of the materials used in this study.

Specimen number	Animal	Sex	Age (year)	Body length (cm)	Body weight (kg)	Time from death to sampling (h)	Origin	Date of death	Examinations	Tissue sample size (depth (cm) × wide (cm))
SNH11012	Pacific white-side dolphin (*Lagenorhynchus obliquidens*)	F	2	143	N/A	>48	Incidental catch in Northwest Pacific Ocean	20. May.11 (presumption)	Histology, CT	3 × 4
UMUT-12343	Bottlenose dolphin (*Tursiops truncatus*)	F	>16	281.5	194	36	Enoshima aquarium, Kanagawa, Japan	19.May.12	Histology, WB	5 × 5
EL-13175	Bottlenose dolphin (*Tursiops truncatus*)	M	2	247.8	162	24	Enoshima aquarium, Kanagawa, Japan	2.Dec.13	Histology, CT	4 × 6
UMUT-14006	Bottlenose dolphin (*Tursiops truncatus*)	F	2 weeks	123.6	21	48	Enoshima aquarium, Kanagawa, Japan	12.Aug.13	Histology, WB, CT	1 × 3
EL-13137	Bottlenose dolphin (*Tursiops truncatus*)	F	Stillbirth	124.2	18	48	Enoshima aquarium, Kanagawa, Japan	25.Jul.13	Histology, WB	1 × 3
SNH11011	Dall's porpoise (*Phocoenoides dalli*)	F	4	174.5	N/A	>48	Incidental catch in Northwest Pacific Ocean	19. May. 11 (presumption)	Histology, CT	3 × 3
SNH13005-1	Harbour porpoise (*Phocoena phocoena*)	M	6	147.0	44.2	>48	Incidental catch in Hokkaido, Japan	17. Apr. 13 (presumption)	Histology, WB	3 × 3
SNH13005-2	Harbour porpoise (*Phocoena phocoena*)	F	2	141.3	40.3	>48	Incidental catch in Hokkaido, Japan	17. Apr. 13 (presumption)	Histology, WB	3 × 3
SNH10020	Harbour porpoise (*Phocoena phocoena*)	M	N/A	139.4	N/A	>48	Incidental catch in Hokkaido, Japan	6. May. 10 (presumption)	CT	N/A

**Fig 1:**
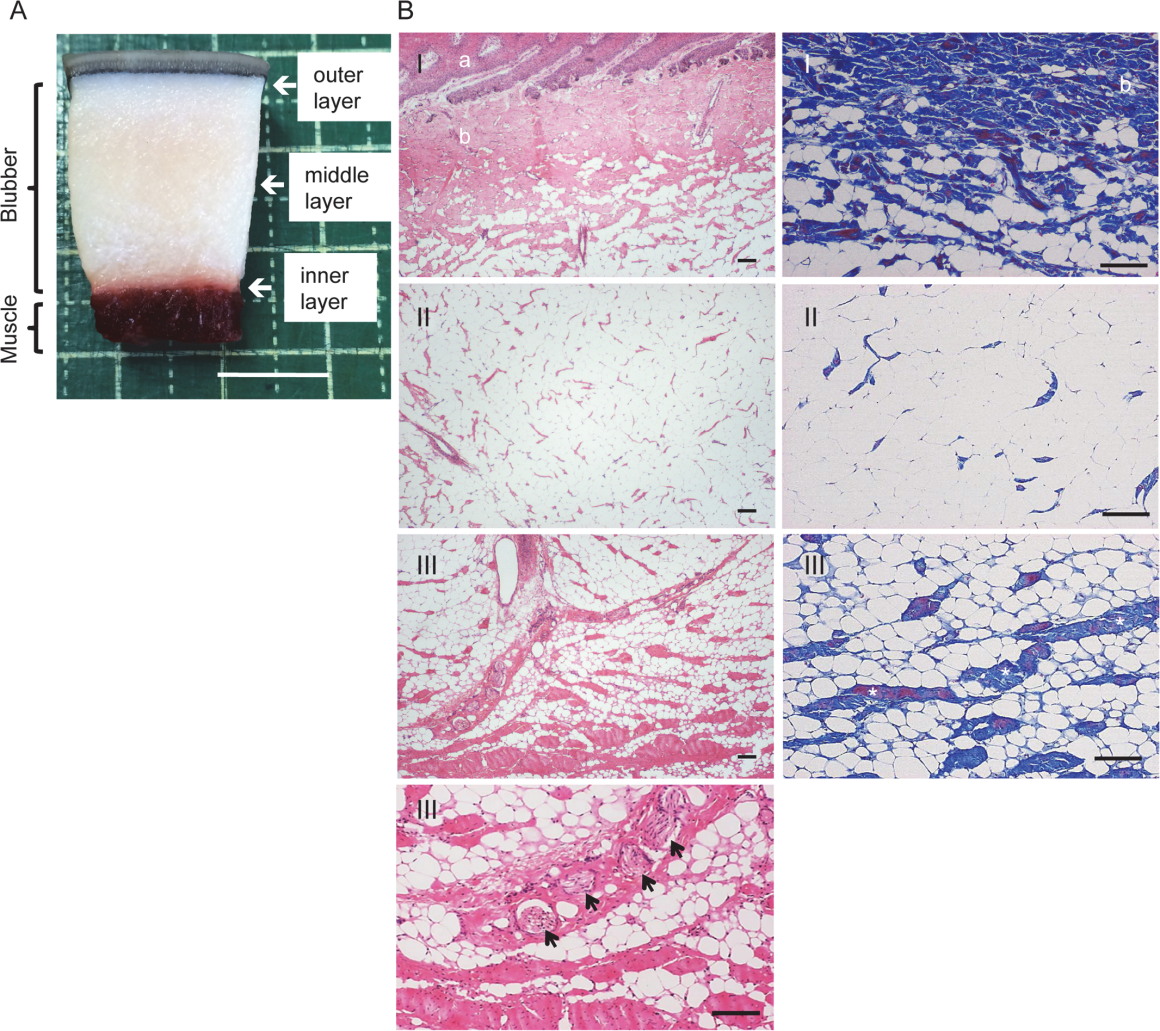
Histology of dolphin blubber. (A) Representative tissue sample taken for histology (EL-13175). Scale bar = 1 cm. (B) Pacific white-side dolphin (SNH11012) blubber, fixed, sectioned and stained (HE, Left panel; Azan, Right panel): outer (I), middle (II) and inner (III) layer of the blubber. a, epidermis; b, dermis. Connective tissues indicated by asterisks. Nerve fibres indicated by arrows. Scale bar = 100 μm.

Full-depth integumental samples including skin, blubber and a part of muscle (Fig. 1A) were taken from a lateral region on the mid-thoracic side of each carcass and either fixed immediately for histological analysis or frozen at −20°C until use. Tissue samples from the lateral region of the abdomen or gluteal dorsal site were also taken from some specimens (SNH11012, UMUT-14006, EL13137, SNH13005-1, SNH13005-2). At least two tissue blocks were taken from each sampling site.

### Ethics statement

No invasive methods were used in this study. All samples were collected from animals that were already deceased. All animals either accidentally died in fishing operations or had died in an aquarium; none was sourced from any targeted cetacean fishery. This study was conducted in accordance with the requirements of the Ethics committee of Kitasato University School of Veterinary Medicine.

### Histological analysis

Tissues were fixed in Bouin's fluid and embedded in paraffin. Sections of 4 μm were affixed to slides, deparaffinized and stained with haematoxylin and eosin (HE) stain for cell structure or Azan stain for connective tissue. Three regions were recognized in each blubber section: outer, middle and inner layers. Within each layer, fat droplet and adipose cell cytoplasm size were measured in three randomly selected fields (0.36 mm^2^ per area) using ImageJ 1.37v. Results are expressed as means ± SEM. Tukey-Kramer multiple comparison tests using a Prism5 software (GraphPad software Inc., San Diego, CA, USA) were used to compare results between layers of blubber. *P* < 0.05 was considered statistically significant. The number of nerve fibre bunches in the blubber was counted within three randomly selected fields of approximately 1 cm^2^.

For immunohistochemistry, deparaffinized sections were incubated with H_2_O_2_, blocked with 10% normal goat serum, then reacted with rabbit polyclonal to UCP-1 antibody (3 μg/ml, No. ab10983, Abcam, Cambridge, UK) or rabbit normal IgG (3 μg/ml, Abcam, No. ab27478) overnight at 4°C, then visualized with 3,3′-diaminobenzidine tetrahydrochloride (DAB) using Histofine Simple Stain MAX-PO kit (Nichirei, Tokyo, Japan), in accordance with earlier described methodology [23].

### Western blotting

Sample preparation was performed as described previously [23]. Tissues from the middle or inner layers of blubber or muscle (approximately 100 mg each) were rinsed in ice-cold PBS and homogenized in 1 ml RIPA lysis buffer supplemented with a protease inhibitor cocktail (Complete Mini, Roche, Indianapolis, IN, USA). The protein content of each extract was determined by protein assay kit (Bio-Rad Laboratories, Inc. Hercules, CA, USA). Proteins (10 μg/lane) were subjected to SDS-PAGE with 12% gel under reducing conditions, then transferred onto polyvinylidene difluoride (PVDF) membranes. Membranes were blocked with 5% non-fat dry milk and probed with anti-UCP-1 antibody (0.1 μg/ml, No. ab10983, Abcam), normal rabbit IgG (0.1 μg/ml, No. ab27478, Abcam), or anti-β-actin antibody (0.1 μg/ml, No. ab8226, Abcam), followed by incubation with a horseradish peroxidase-conjugated secondary antibody. The reaction was detected with a chemiluminescence system (ECL Prime; GE Healthcare, Little Chalfont, UK).

### CT analysis

To better realize the distribution of BAT throughout the cetacean body, a subcutaneous tissue block (~6 × 4 cm, EL-13175) and one entire carcass (UMUT-14006) were scanned by CT (Aquilion 16, Toshiba Medical Systems, Tokyo, Japan), serially from cranial to caudal planes in parallel at 1-mm thickness without a gap. Voxel Transmission (Volume Rendering) techniques then were applied to visualize subcutaneous tissues using a 3D image analysing system (AZE virtual pace, AZE Corporation, Tokyo, Japan). Additional carcasses (SNH11011, SNH11012 and SNH10020) also scanned by CT (Activion 16, Toshiba Medical Systems, Tokyo, Japan) were reconstructed at 2-mm thickness at 1-mm intervals with a soft-tissue filter using OsiriX software (ver. 5.8, 64-bit, The OsiriX Foundation, Geneva, Switzerland).

## Results

Macroscopically the blubber was thick and connected to skeletal muscle. Layers of blubber were defined by their position relative to the skin as outer, middle or inner (Fig. 1A). Histology revealed a thick subcutaneous deposit of blubber surrounding the skeletal muscle. The inner layer of blubber was rich in adipocytes, intermingled with connective tissue and cutaneous muscles and nerve fibres (Fig. 1B, Tables 2–4). In contrast to the adipocytes of the outer and middle layers of blubber, which had large unilocular fat droplets and small cytoplasm, those of the inner layer had small unilocular fat droplets and large eosinophilic cytoplasm (Fig. 1, Tables 2–4).

**Table 2:** Characterization of the adipocytes in cetacean blubbers from thoracic region.

Specimen	Animal	Area of fat droplet /cell in blubber (μm^2^)			Area of cytoplasm /cell in blubber (μm^2^)			Number of nerve fibre bunch in blubber (/ cm^2^)			UCP1 immuno-reaction of the inner layer
		outer layer	middle layer	inner layer	outer layer	middle layer	inner layer	outer layer	middle layer	inner layer	
SNH11012	Pacific white-side dolphin	1563±45	2027±168	1204±131^b^	49.0±1.7	28.3±1.2^a^	69.7±3.0^a^ ^,^ ^b^	1.23	0	9.88	++
UMUT-12343	Bottlenose dolphin	4970±225	5029±423	1369±95^a,^ ^b^	29.6±3.8	29.4±4.0	83.6±12.7^a^ ^,^ ^b^	0	0	1.18	++
EL-13175	Bottlenose dolphin	2981±34	3623±284	2166±198^b^	56.3±8.1	103.0±25.1	217.7±31.4^a^ ^,^ ^b^	0	0	1.18	±
UMUT-14006	Bottlenose dolphin	928±11	1176±52^a^	868±55^b^	56.3±4.9	53.3±3.5	85.7±8.4^a^ ^,^ ^b^	0.96	0	1.92	−
EL13137	Bottlenose dolphin	1187±37	1370±82	793±56^a,^ ^b^	31.7±3.9	32.3±4.2	63.3±7.3^a^ ^,^ ^b^	0	0	3.06	−
SNH11011	Dall's porpoise	1662±103	2899±174^a^	940±59^a^ ^,^ ^b^	68.7±4.2	30.0±1.7^a^	116.0±13.3^a^ ^,^ ^b^	1.59	0.79	2.38	+
SNH13005-1	Harbour porpoise	1544±80	2071±177	1353±199^b^	61.0±4.0	33.7±9.0	178.7±30.3^a^ ^,^ ^b^	0.76	0	2.27	− *
SNH13005-2	Harbour porpoise	1601±27	2260±336	1439±83	52.3±4.8	43.7±9.4	116.0±5.1^a^ ^,^ ^b^	0	0	4.48	− *
SNH10020	Harbour porpoise	N/A	N/A	N/A	N/A	N/A	N/A	N/A	N/A	N/A	N/A

Values are mean ± SEM.

^a^, P < 0.05 vs outer layer;

^b^, P < 0.05 vs middle layer.

The intensity of reaction is given on a 3-point scale: (−) negative, (±) marginal positive, (+) positive, (++) strong positive.

*, The specific band for UCP1 was detected in Western blotting.

**Table 3:** Characterization of the adipocytes in cetacean blubbers from abdominal region.

Specimen	Animal	Area of fat droplet /cell in blubber (μm^2^)			Area of cytoplasm /cell in blubber (μm^2^)			Number of nerve fibre bunch in blubber (/ cm^2^)			UCP1 immuno-reaction of the inner layer
		outer layer	middle layer	inner layer	outer layer	middle layer	inner layer	outer layer	middle layer	inner layer	
SNH11012	Pacific white-side dolphin	1588±163	2437±105^a^	577±32^a^ ^,^ ^b^	66.7±1.2	25.3±3.5^a^	69.7±1.5^b^	0	0	2.9	++
UMUT-12343	Bottlenose dolphin	N/A	N/A	N/A	N/A	N/A	N/A	N/A	N/A	N/A	N/A
EL-13175	Bottlenose dolphin	N/A	N/A	N/A	N/A	N/A	N/A	N/A	N/A	N/A	N/A
UMUT-14006	Bottlenose dolphin	725±42	957±13^a^	571±62^b^	63.3±13.4	46.3±2.4	85.7±11.5	0	0	5.81	−
EL13137	Bottlenose dolphin	885±31	1100±26^a^	246±9^a^ ^,^ ^b^	39.0±4.4	25.7±3.3	76.0±1.0^a^ ^,^ ^b^	0	0	1.5	−
SNH11011	Dall's porpoise	N/A	N/A	N/A	N/A	N/A	N/A	N/A	N/A	N/A	N/A
SNH13005-1	Harbour porpoise	1025±30	2480±34^a^	945±66^b^	41.0±5.2	23.3±1.2^a^	62.0±1.5^a^ ^,^ ^b^	0.56	0	1.12	−
SNH13005-2	Harbour porpoise	1242±44	3411±770^a^	1039±128 ^b^	34.0±3.2	32.3±2.0	91.3±2.3^a^ ^,^ ^b^	0	0	1.63	−
SNH10020	Harbour porpoise	N/A	N/A	N/A	N/A	N/A	N/A	N/A	N/A	N/A	N/A

Values are mean ± SEM.

^a^, P < 0.05 vs outer layer;

^b^, P < 0.05 vs middle layer.

The intensity of reaction is given on a 3-point scale: (−) negative, (±) marginal positive, (+) positive, (++) strong positive.

**Table 4:** Characterization of the adipocytes in cetacean blubbers from caudal region.

Specimen	Animal	Area of fat droplet /cell in blubber (μm^2^)			Area of cytoplasm /cell in blubber (μm^2^)			Number of nerve fibre bunch in blubber (/ cm^2^)			UCP1 immuno-reaction of the inner layer
		outer layer	middle layer	inner layer	outer layer	middle layer	inner layer	outer layer	middle layer	inner layer	
SNH11012	Pacific white-side dolphin	N/A	N/A	N/A	N/A	N/A	N/A	N/A	N/A	N/A	N/A
UMUT-12343	Bottlenose dolphin	N/A	N/A	N/A	N/A	N/A	N/A	N/A	N/A	N/A	N/A
EL-13175	Bottlenose dolphin	N/A	N/A	N/A	N/A	N/A	N/A	N/A	N/A	N/A	N/A
UMUT-14006	Bottlenose dolphin	669±31	748±53	440±2^a^ ^,^ ^b^	38.3±5.2	40.3±2.4	59.7±5.4^a^	1.12	0	2.25	−
EL13137	Bottlenose dolphin	885±41	1449±42^a^	227±12^a^ ^,^ ^b^	48.0±5.1	31.7±2.0^a^	51.0±2.1 ^b^	0	0	6.25	−
SNH11011	Dall's porpoise	N/A	N/A	N/A	N/A	N/A	N/A	N/A	N/A	N/A	N/A
SNH13005-1	Harbour porpoise	1509±25	2558±173^a^	1210±36^b^	42.3±3.2	24.3±1.5	98.3±8.6^a^ ^,^ ^b^	0.25	0	1.27	−
SNH13005-2	Harbour porpoise	1382±80	2928±146^a^	1025±80 ^b^	48.3±4.2	43.7±2.7	81.7±8.2^a^ ^,^ ^b^	0	0	1.65	−
SNH10020	Harbour porpoise	N/A	N/A	N/A	N/A	N/A	N/A	N/A	N/A	N/A	N/A

Values are mean ± SEM.

^a^, P < 0.05 vs outer layer;

^b^, P < 0.05 vs middle layer.

The intensity of reaction is given on a 3-point scale: (−) negative, (±) marginal positive, (+) positive, (++) strong positive.

**Fig 2:**
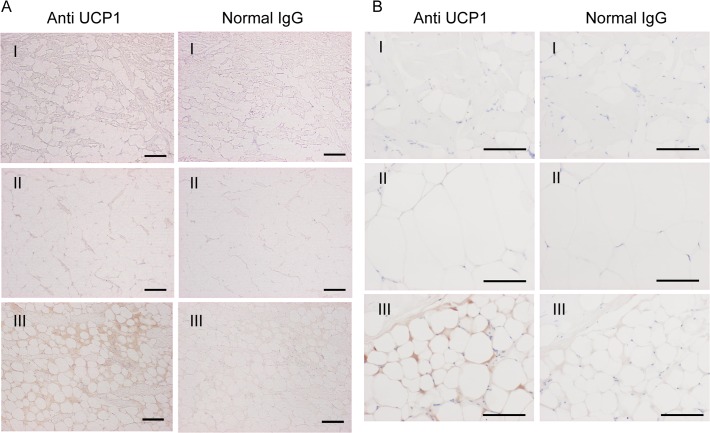
Immunohistochemical analysis of dolphin blubber. Sections of blubber from (A) Pacific white-side dolphin (SNH11012) and (B) bottlenose dolphin (UMUT-12343), immunoreacted with anti-UCP1 antibody or normal IgG. Outer (I), middle (II) and inner (III) layer. Scale bar = 100 μm.

Immunohistochemical analysis using anti-UCP1 antibody revealed a concentration of brown adipocytes with a small fat droplet and large cytoplasm in the inner layer of blubber (Fig. 2); UCP1-positive cells were not observed in other blubber layers (Fig. 2). Reactivity decreased with normal rabbit IgG primary antibody (Fig. 2). Immunoreactivity of UCP1 in the innermost blubber layer varied between specimens (Tables 2–4), possibly because of differences in sample freshness (where known, tissues were collected from 24–48 hours post mortem). Alternatively, UCP1 expression may have been influenced by the initial health or condition of the specimen or the water temperature in which it lived.

**Fig 3:**
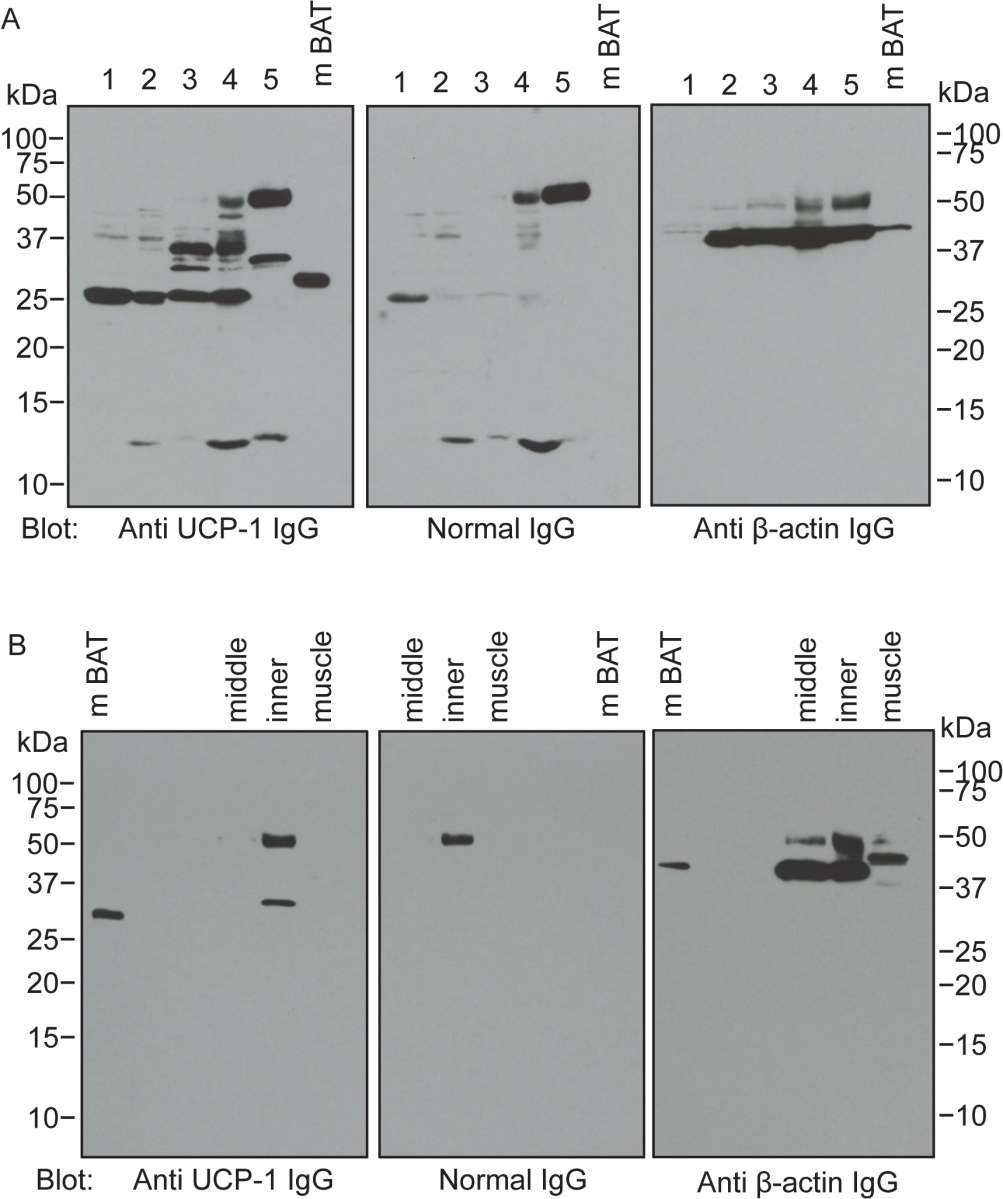
Western blot analysis of lowest layer of delphinoid blubber. Tissue extracts subjected to SDS-PAGE immunoblotting analysis using anti-UCP1, normal IgG or β-actin antibodies. (A) The blots of inner blubber layer extracts from cetaceans. 1, EL-13137; 2, UMUT-14006; 3, SNH13005-1; 4, SNH13005-2; 5, UMUT-12343. (B) The blots of middle, inner blubber layer and muscle extracts from UMUT-12343. Mouse BAT extracts were loaded as a positive control (mBAT). Data are representative of three separate experiments. Protein migration indicated by bars to the left and right of the figure.

Western blot analysis of extracts from the inner blubber layer confirmed the immunohistochemical positive reaction to be specific to UCP1. Anti-UCP1 antibody reacted with various proteins of molecular weight ranging from 12–50 kDa (Fig. 3A). The 34−36-kDa bands (unique to anti UCP1 rabbit IgG) were likely specific for delphinoid UCP1. Fig. 3B depicts the positive reaction of the UCP1 antibody specific to the inner blubber layer.

**Fig 4:**
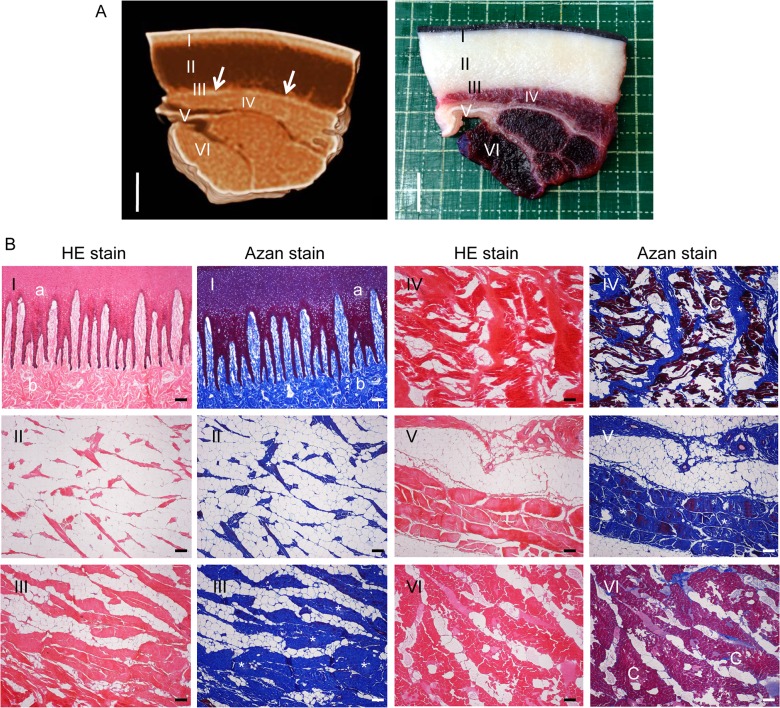
Detection of brown adipose tissue in dolphin blubber by CT scanning. (A) CT observation of bottlenose dolphin blubber block (EL-13175) with thin, highly dense layer indicated by arrows (left panel). Cross section of the block roughly corresponding to the CT image (right panel). Scale bar = 1 cm. (B) Sampling sites for histology labelled I–VI in A. Left panel, HE stain. Right panel, Azan stain. a, epidermis; b, dermis; c, skeletal muscle. Connective tissues indicated by asterisks.

**Fig 5:**
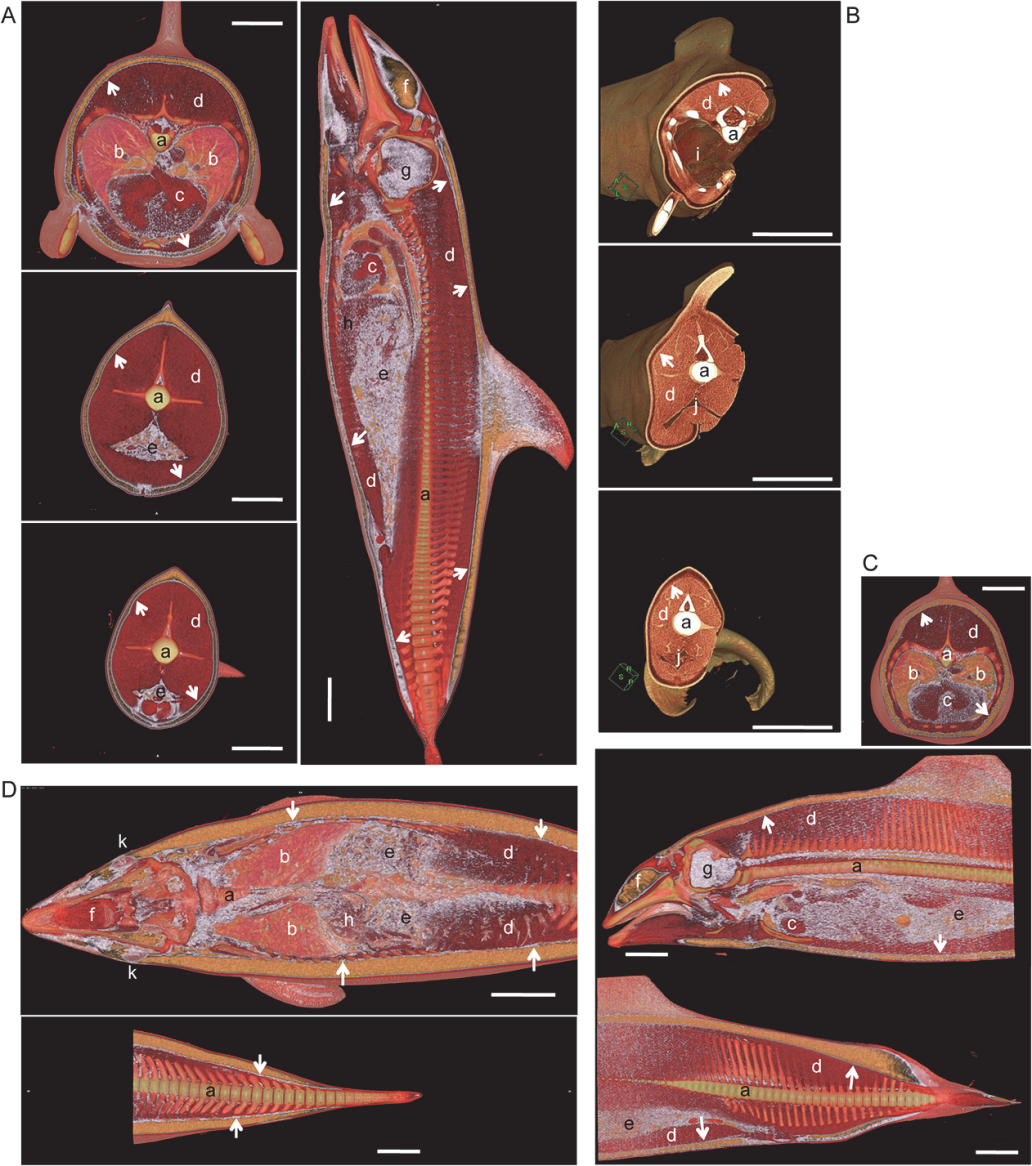
Distribution of brown adipose tissue in cetacean blubber. (A) Whole body CT of Pacific white-side dolphin (SNH11012). Thoracic region (left upper panel), Abdominal region (left middle panel), Tail region (left lower panel). Lateral view of the body (right panel). (B) Whole body CT of bottlenose dolphin (UMUT-14006). The animal was scanned after necropsy. Thoracic region (upper panel), Abdominal region (middle panel), Gluteal region (lower panel). (C) Whole body CT of Dall's porpoise (SNH11011). Thoracic region (upper panel). Lateral view of the body (lower panel). (D) CT of Harbour porpoise (SNH10020). Horizontal section of the body. Arrows: thin layer of high-density/connective tissues in lower blubber layer. a, vertebrae; b, lung; c, heart; d, skeletal muscle; e, intestine; f, melon; g, brain; h, liver; i, thoracic cavity; j, abdominal cavity; k, eye. Scale bar = 10 cm.

CT revealed a thin layer of high-density tissue between the blubber and skeletal muscle (Fig. 4A), which histology confirmed to be connective tissue in the inner layer of blubber (Fig. 4B). This layer extended the greater length of the dolphin body, with the exception of the rostrum, fins and flukes (Fig. 5). Hounsfield scale values within this thin layer were −30 to −10 HU, higher than those of general adipose tissue (approximately −70 to −60 HU), but lower than those of soft tissue (around 30 HU).

## Discussion

It has been assumed that cetaceans swim continuously, even during their sleep [24], to maintain body temperature. However, long periods of inactivity have been reported for captive bottlenose dolphins [25], the body temperature of which correlated negatively with water temperature and positively with serum noradrenalin and adrenalin concentration [26]. How these animals regulated their body temperature in cool waters during periods of prolonged inactivity was a mystery. Our thesis was that thermogenesis by BAT occurred among these dolphins during these inactive periods, though until now BAT was unknown from cetaceans. We detected UCP1 expression in the blubber of two species each of dolphin and porpoise using a combination of techniques (histological, biochemical, and computerized tomography), and showed it to be distributed the length of the body, with the exception of the rostrum, fin and fluke regions. We believe delphinoid BAT existed in the inner layer of blubber and that it might play some role in regulating body temperature.

The detection of UCP1, a brown adipocyte-specific mitochondrial protein in adipose tissues, is useful for identifying BAT, though it is difficult to detect in old (not fresh) samples. Furthermore, UCP1 expression may have been influenced by the initial health condition of the specimen or the water temperature in which it lived. Therefore, in addition to determining UCP1 expression, we also investigated fat droplet and cytoplasm size in order to detect brown adipocytes in available blubber samples. The combined results suggest that BAT was distributed in the inner layer of blubber. Nerve fibres within the inner layer of delphinoid cetacean blubber innervate BAT (UCP1-positive adipose tissues), in contrast to those of the outer and middle layers. We suggest that such innervated tissue is directly involved in thermoregulation in these cetacean taxa in a manner similar to that in comparably innervated rat tissue [27]. We also suggest that, at least in these studied cetaceans, blubber acts not only as a conventional blanket in minimizing heat loss, but also as an electric blanket to generate heat.

Histologically, brown adipocytes in the inner layer of the blubber were associated with connective tissue. CT revealed such connective tissue to correspond to a thin and highly dense layer in the blubber, which occurred throughout the cetacean body, suggesting the trunk of the body was wrapped in BAT. Although a similar layer was apparent between the skin and blubber (Fig. 5), no brown adipocytes were present within it (Fig. 2, Tables 2–4), and as such it does not constitute BAT. Thin, highly dense layers were also observed in the gluteal region, but given the absence of cutaneous muscles these layers do not correspond to ordinary connective tissue like fascia. Furthermore, the Hounsfield scale values of cutaneous muscle with fascia (> 30–40 HU) differ from the thin layer in the inner layer of the blubber.

There is variation in the nature of fat droplets in BAT tissues among those animals from which it has been described. Fat droplets of smaller brown and beige/brite adipocytes in rodents are typically multilobulated [2], whereas those of harp seals, have brown adipocytes with multilocular fat droplets [28]. The UCP1 positive adipocytes in delphinoid cetaceans described here are unilobulated like those of cattle [29]. These fat droplets can also vary within a taxon: PET-CT and histological analysis has even revealed that cold exposure induced human supraclavicular BAT comprises cells of both a multilobulated and unilobulated fat droplet nature [30]. These human BAT are a mixture of brown and brite/beige cells that equally express UCP1 and contribute to thermogenesis [31, 32]. Further study is necessary to determine cetacean BAT origins, its ubiquity in the group, and the significance, if any, of the shape of the fat droplets.

Harp seal pups between 0 and 7 days old increased their O_2_ consumption when exposed to the cold, but older pups did not [33], suggesting either a physical or functional loss of BAT over time; UCP1 expression in BAT from the neck region of harp seals has also been described as decreasing during growth [34]. That we recognize BAT in adults of at least four delphinoid taxa, Dall’s porpoise, harbour porpoise and bottlenose and white-sided dolphins (Table 2, Fig. 3), suggests this tissue might be more widely distributed among aquatic mammals than currently recognized, and that the mechanisms of BAT thermoregulation may vary not only between taxa, but in different life history stages of a single taxon. Additionally, as cetaceans are obligate aquatic mammals that lack fur, their mechanisms of BAT heat regulation might differ from seals.

The mouse has two putative *N*-glycosylation sites in UCP1 proteins (GenBank accession number, NM_009463), whereas cattle (NM_001166528) and sheep (JN604984), more closely related to cetaceans than to the mouse, have three. Therefore, it seems that the UCP1 positive bands detected at higher molecular weight compared with mouse UCP1 are heavy glycosylation forms. The nucleotide sequence and molecular characteristics of the dolphin UCP1 gene and the function of the sugar chain remain to be determined.

In summary, this is the first study to report BAT in the blubber of any cetacean taxon, and we report it from distantly related porpoises and dolphins. We describe adipocytes as having small unilocular fat droplets and a large eosinophilic cytoplasm, distributed throughout a thin and highly dense layer that extends much of the length of the delphinoid body, excluding the rostrum, fin and fluke regions. Our results suggest this inner layer of blubber is capable of performing a role analogous to that of an electric blanket, separate from any other more universally accepted role blubber might play in basic insulation, storage of energy reserves, hydrodynamic streamlining, and/or buoyancy control. The existence of BAT in deeper-diving, higher-latitude, and/or more extensively migratory and non-delphinoid cetacean taxa, or throughout the life cycle of any given delphinoid taxon, remains to be demonstrated.
